# Correction to: ‘Towards a system-level causative knowledge of pollinator communities’ 2022 by Saavedra *et al.*

**DOI:** 10.1098/rstb.2022.0453

**Published:** 2023-02-13

**Authors:** Serguei Saavedra, Ignasi Bartomeus, Oscar Godoy, Rudolf P. Rohr, Pengjuan Zu

*Phil. Trans. R. Soc. B* **377**, 20210159. (Published online 2 May 2022). (https://doi.org/10.1098/rstb.2021.0159)

In the original version of this article, author Pengjuan Zu's name was spelled incorrectly.

This has now been corrected on the publisher's website.

